# Conservative Treatment of Uterine Cervical Adenocarcinoma in Pregnancy

**DOI:** 10.1155/2013/692017

**Published:** 2013-12-17

**Authors:** Cid Almeida de Lima, Ana Cristina Macêdo Barcelos, Marina de Carvalho Paschoini, João Henrique do Amaral e Silva, Marcus Aurelho de Lima, Eddie Fernando Candido Murta, Rosekeila Simões Nomelini

**Affiliations:** ^1^Research Institute of Oncology (IPON)/Discipline of Gynecology and Obstetrics, Federal University of Triângulo Mineiro (UFTM), Averida Getúlio Guaritá, 214, Bairro, 38025-440 Uberaba, MG, Brazil; ^2^Discipline of Gynecology and Obstetrics, Federal University of Triângulo Mineiro (UFTM), Uberaba, MG, Brazil; ^3^Service of Surgical Pathology, Federal University of Triângulo Mineiro (UFTM), Uberaba, MG, Brazil; ^4^Pathology Service Patmed, Association Against Cancer in Central Brazil, Uberaba, MG, Brazil

## Abstract

Cancer of the cervix is the most common malignancy diagnosed during pregnancy, with an incidence of 1–10 cases per 10,000 pregnancies. The desire of patients to maintain pregnancy and subsequent fertility is a difficult target to be achieved and should be widely studied, since it depends on the stage of disease, gestational age at diagnosis, and the woman's desire to maintain pregnancy. We describe in this report the case of a pregnant woman with invasive cervical adenocarcinoma in stage IB1 (FIGO) initially treated with neoadjuvant chemotherapy, followed by radical surgery and cesarean section in the same surgical procedure.

## 1. Introduction

Early diagnosis and treatment of patients with cervical cancer have advanced greatly in recent years, but many women in the world still suffer from this disease and many are in reproductive age [1]. The cervical cancer is a malignant tumor most commonly diagnosed during pregnancy [2, 3] and its estimated incidence is of 1–10 per 10,000 pregnancies [2, 4]. Nevertheless, there is no protocol defined for the conduct of these cases [5] and studies in this area are extremely important [1].

The management of cervical cancer diagnosed during pregnancy requires complex clinical and ethical decisions [4] and represents a challenge for clinicians [2]. For many authors, conducting depends on the stage of the disease, gestational age at diagnosis, and the woman's desire to maintain pregnancy [2, 4, 6–8]. In pregnancies, initial standard treatment requires termination of pregnancy [4, 9] and loss of reproductive capacity [9]. Cesarean section and definitive treatment of the mother are mainly carried out in the near-term fetuses [4].

Due to cultural and ethical dilemmas inherent in this situation [7], some researchers have published cases of patients where treatment was intentionally delayed to await fetal maturity [2, 7]. We describe in this report the case of a pregnant woman with invasive carcinoma of the cervix, stage IB1 (FIGO), initially treated with neoadjuvant chemotherapy, followed by caesarean section and radical hysterectomy.

## 2. Case Report

**Figure 1:**
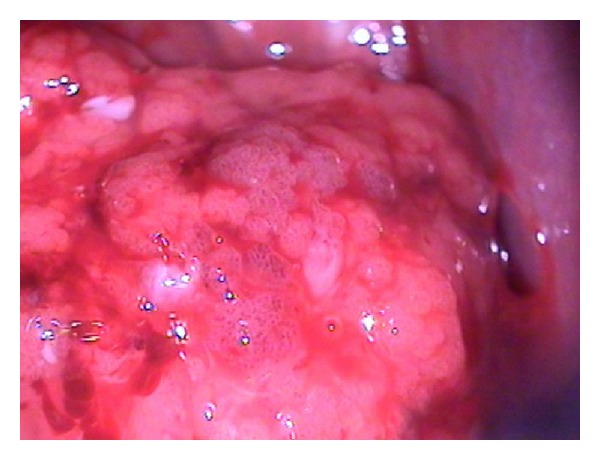
Colposcopy: cerebroid lesion with dense acetowhite epithelium, mosaic, and atypical vascularization.

A 24-year-old woman in the 23rd gestational week, gravida 5, para 1, sought obstetrics service referring aqueous discharge with odor amount for 4 weeks. The gynecologic examination demonstrated friable and cerebroid lesion easily bleeding, externalizing the external orifice of the uterine cervix, and measuring approximately 4 cm. The colposcopic evaluation showed dense acetowhite epithelium and mosaic in anterior lip of the cervix with atypical vascularization and papillary acetowhite lesion externalizing the cervix (Figure 1). Biopsy of the lesion showed histological diagnosis of invasive moderately differentiated mucinous adenocarcinoma of the endocervical type.

**Figure 2:**
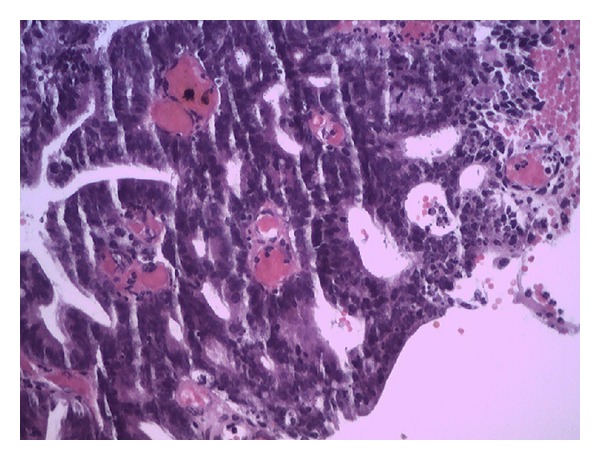
Invasive moderately differentiated mucinous adenocarcinoma endocervical type composed of irregular and anastomosing glands with cribriform arrangements.

**Figure 3:**
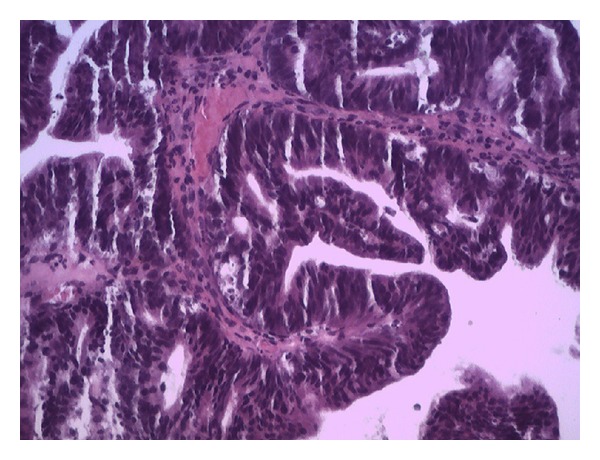
Invasive moderately differentiated mucinous adenocarcinoma endocervical type composed of irregular and anastomosing glands with papillary arrangements.

The pathological study showed malignant epithelial neoplasm which is composed of glands of irregular shapes, anastomosing with cribriform (Figure 2) and papillary (Figure 3) arrangements whose cells have a cylindrical shape with a high ratio nucleus/cytoplasm, hyperchromatic nuclei, with evident nucleoli, frequent mitotic figures, and vacuolated cytoplasm. In light of the glands, there is cellular debris. The stroma between the cells is scarce and is represented by desmoplastic fibrous connective tissue. The research of mucus, by Alcian blue technique, was positive.

The tumor stage was IB1 (FIGO). Pelvic magnetic resonance imaging (MRI) confirmed the presence of a cervical lesion and no involvement of parametria, measuring 3.2 cm × 2.1 cm × 2.5 cm.

Pregnant woman and her family were informed of the diagnosis and therapeutic possibilities. The patient refused to end pregnancy for treatment of malignancy which proposed neoadjuvant chemotherapy with cisplatin (50 mg/m^2^) plus vincristine (1 mg/m^2^) administered every 4 weeks between 26- and 30-week gestation (two cycles). There was partial regression of the lesion. Corticosteroid (betamethasone) was administered in order to accelerate fetal lung maturity. At a 34-week gestation, a caesarean section followed by radical hysterectomy and pelvic lymphadenectomy (Wertheim-Meigs surgery) was performed. The infant was a female weighing 2150 g with an Apgar score at 1 and 5 minutes of 9 and 9.

Postsurgical specimen showed cervical villoglandular adenocarcinoma, measuring 3 cm × 2 cm × 1.7 cm and infiltrating superficially the connective axis of a polyp with foci of adenocarcinoma *in situ* and cervical intraepithelial neoplasia 3 (CIN 3). CIN 3 and adenocarcinoma *in situ* were not found in the initial biopsy. This could be explained by the small fragment of incisional biopsy, while the entire surgical specimen was evaluated postoperatively. Nine pelvic lymph nodes were dissected and were without cancer. The patient underwent further 4 cycles of chemotherapy. The patient presents with no signs of tumor recurrence two years after initial diagnosis.

## 3. Discussion

There are some possibilities for the treatment of invasive cervical cancer during pregnancy with the chance to keep the pregnancy. The first is the postponement of radical surgery (with or without subsequent adjuvant therapy) until fetal maturity, when increase the chances of survival of the fetus outside the uterus [2, 4, 5]. This treatment is limited to women with initial staging and in the second or third trimesters of pregnancy [4]. Another option for patients who refuse to end pregnancy is neoadjuvant chemotherapy [1, 8]. This treatment is performed to improve the prognosis of this disease [6], through elimination of micrometastases and primary tumors decreasing the surgical preparation of the patients [1, 4], while awaiting the fetus to mature.

The cervical carcinoma is one of the most common cancers during pregnancy. It was observed that more than 50% of cervical cancers were found in the postpartum period, 30% at birth, and only 20% in the prenatal [1]. In the present report, the diagnosis was made in the prenatal period, in the 23rd week of pregnancy, where the patient chose to continue the pregnancy.

The desire to maintain the pregnancy, despite a malignant disease, is a challenge [2] and requires complex medical and ethical decisions [3]. The best approach to be adopted involves factors such as gestational age at diagnosis, stage of disease, and the patient's desire to maintain pregnancy [4].

In this study, neoadjuvant chemotherapy was performed [10]. Counseling should include the potential effects of fetal treatment. During the first trimester, the estimated risk teratogenic to the fetus varies from 7.5 to 17%, when a single agent is administered, and increases to 25% when combination chemotherapy is used [11]. Moreover, the risk of birth defects when cytotoxic drugs are administered during the second and third trimesters is similar to that of the general population (1–3%). The main fetal effects of chemotherapy during the second and third trimesters relate to growth restriction intrauterine fetal death intrauterus, prematurity, and low birth weight [12]. These precepts are based on reported cases, and the treatment should be carefully individualized and extensive advice should be provided to the patient and her family [9]. Our patient has signed the written consent form before starting chemotherapy.

Mandic et al. described a patient undergoing neoadjuvant chemotherapy with continuation of pregnancy without cervical carcinoma recurrence after 6 months of postnatal follow-up. In addition, no abnormality was detected in children [2]. Delaying radical surgery improves fetal maturity, increasing the chances of the fetus surviving outside the uterus [6]. Still this type of treatment is limited to patients in stage I cervical carcinoma, second or early third trimester of pregnancy [7]. According to the literature, radical surgery is postponing conduct of more acceptance and more reasonable treatment in stage I cancer of the cervix in pregnant patients [4, 13–15]. Some studies suggest the trachelectomy as a way to maintain fetal viability in young pregnant women who wish to maintain fertility [16]. However, in these cases, the fetal loss may be inevitable [17].

This report demonstrates that a conservative strategy and noninvasive treatment in cervical carcinoma can be applied in carefully selected patients with early stage cervical cancer diagnosed during pregnancy. The treatments described in the literature must be studied better, since there are still disagreement methods of treatments.
